# The Book World of Medicine and Science

**Published:** 1894-08-04

**Authors:** 


### THE BOOK WORLD OF MEDICINE AND SCIENCE.

### BOOK REVIEWS.

The Official Year-Book of the Scientific and Learned Societies of Great Britain and Ireland. (London: Charles Griffin and Company, Limited.)

The issue of this number marks the completion of the first decade in the life of a most useful book of reference. As its title implies, the work is a handbook which records the transactions and proceedings of the various scientific and learned societies in the United Kingdom, and it is a step towards the centralisation of scientific records which Professor Michael Foster so strongly advocated at the International Medical Congress held this year at Rome. Original workers in the field of science find so many difficulties in keeping pace with the publication of papers, even on their own special subject, that it is hardly remarkable when, as so often happens, independent workers publish papers on one and the same subject in the transactions or proceedings of private societies, in ignorance that they are traversing old ground. The possession of the "Year-Book" and its careful perusal will do much to minimise disputes between claimants for the priority of discovery or publication, and direct into useful channels much energy which is often, through ignorance, squandered in repeating old work. Men of science and scientific societies generally owe a debt of gratitude to Messrs. Griffin for the publication of the "Year-Book."

Pharmacopœia of the National Hospital for Diseases of the Heart. (London: John Bale and Sons.)

This little pharmacopœia has been drawn up by the staff of the hospital. It contains some two or three hundred useful prescriptions, not only such as are of use in cardiac cases, but also such as are of general application in medicine. The interpolation of plain sheets between the printed matter is a useful adjunct. An alphabetical list of the drugs in common use, with their doses, usually forms a part of a hospital pharmacopœia. No doubt there is some good reason for its omission in this instance.

Curtice's Index to the "Times," London Morning and Evening Papers, &c. (Edward Curtice, 221, High Holborn. 1894.)

The first volume of Mr. Curtice's "Newspaper Index" which lies before us seems to be compiled upon a plan which is at once simple and comprehensive. The field covered is very large, the most important weekly as well as provincial newspapers being included, the volume containing altogether 40,000 indices on every variety of matter. Mr. Curtice is to be congratulated on having produced a reference book which will be of much value to many workers in the political and literary world. The volumes are to be published quarterly, the subscription for the whole year being £2.

Mountain, Moor, and Loch. Illustrated by Pen and Pencil. (Sir Joseph Causton and Sons.)

This is a guide book, but a guide book of the most delightful description. The route described is that traversed by the West Highland Railway. A perusal of the letterpress, a glance at the delightful little studies of the exquisite spots delineated profusely through the pages of this book, is enough to make the traveller forsake all other railways, and indeed all other countries, for Scotland. The West Highland Railway is only opened this month, but if "Mountain, Moor, and Loch" falls into the hands of many tourists the opening session cannot fail to be a brilliant one. We cannot do better than advise intending visitors to Scotland to get this book, as it cannot fail to show them where to go and what to see in the most charming manner and to the best advantage.

The South African Empire. (14, Cockspur Street, S.W.)

The birth roll of new journals is ever growing longer, and with most of the new arrivals extreme youth is the most brilliant period. Progress and improvement does not seem to be a favourite motto. The policy of "The South African Empire" is, however, of a higher order. This well-established colonial paper has blossomed out into a second spring, and is now a veritable box of Pandora, laden with news and subjects likely to interest the New Empire in the Old. Like The Hospital, it studies the wants of its public, and, like The Hospital, it aims at a place in the vanguard of journalism.

